# Identification of Noise Covariance Matrices to Improve Orientation Estimation by Kalman Filter

**DOI:** 10.3390/s18103490

**Published:** 2018-10-16

**Authors:** Alexis Nez, Laetitia Fradet, Frédéric Marin, Tony Monnet, Patrick Lacouture

**Affiliations:** 1 Prime Institute, CNRS-University of Poitiers-ENSMA, UPR 3346, Robotics, Biomechanics, Sport and Health Team, 86360 Chasseneuil du Poitou, France; alexis.nez@ac-reims.fr (A.N.); tony.monnet@univ-poitiers.fr (T.M.); patrick.lacouture@univ-poitiers.fr (P.L.); 2 Laboratoire de Biomécanique et Bioingénierie, UMR CNRS 7338, Université de Technologie de Compiègne, 60203 Compiègne, France; frederic.marin@utc.fr

**Keywords:** inertial sensors, human motion analysis, Kalman filter, covariance matrices, orientation measurement

## Abstract

Magneto-inertial measurement units (MIMUs) are a promising way to perform human motion analysis outside the laboratory. To do so, in the literature, orientation provided by an MIMU is used to deduce body segment orientation. This is generally achieved by means of a Kalman filter that fuses acceleration, angular velocity, and magnetic field measures. A critical point when implementing a Kalman filter is the initialization of the covariance matrices that characterize mismodelling and input error from noisy sensors. The present study proposes a methodology to identify the initial values of these covariance matrices that optimize orientation estimation in the context of human motion analysis. The approach used was to apply motion to the sensor manually, and to compare the orientation obtained via the Kalman filter to a measurement from an optoelectronic system acting as a reference. Testing different sets of values for each parameter of the covariance matrices, and comparing each MIMU measurement with the reference measurement, enabled identification of the most effective values. Moreover, with these optimized initial covariance matrices, the orientation estimation was greatly improved. The method, as presented here, provides a unique solution to the problem of identifying the optimal covariance matrices values for Kalman filtering. However, the methodology should be improved in order to reduce the duration of the whole process.

## 1. Introduction

In the fields of medicine and biomechanics, human motion analysis is often used to extract quantitative and objective data to characterize a movement or a function of the neuro-musculo-skeletal system. During human motion analysis, the aim is frequently to define a body segment rotation with respect to its proximal segment [1]. To perform this analysis, optoelectronic systems that measure the 3D coordinates of reflective markers remain the “gold standard” [1,2]. However, such systems suffer from four main drawbacks: (a) even if such systems could be movable, because cameras have to be handled with caution, most systems are fixed inside the laboratory walls; (b) measurement volume is restricted to the camera’s field of vision; (c) sessions with multiple subjects require numerous cameras to counteract occlusion by the marker; (d) calibration of the whole system can be time-consuming and must be redone if a camera has been moved.

In this context, magneto-inertial measurement units (MIMUs) are a promising, emerging way to perform human motion analysis outside the laboratory, because they are wearable, light, and wireless [3,4]. These MIMUs typically include a combination of 3D accelerometers, gyroscopes, and magnetometers. However, MIMUs do not measure orientation directly. Rather, they measure its linear acceleration, angular velocity, and the magnetic field surrounding the sensor. Angular velocity can be numerically integrated to obtain rotation, but MIMUs designed for human motion analysis are based on low-cost microelectromechanical technology that also suffers from substantial noise and bias over the measurement [5]. This noise and bias particularly affects the angular velocity measure, [6] such that the estimation of the orientation by numerical integration leads to a well-known error: orientation drift [7]. To improve the orientation calculation, the sensor’s data are then combined [8], the linear acceleration and the magnetic field being used to correct the initial estimate obtained by numerical integration of the angular velocity [7]. Indeed, in the absence of movement, the linear acceleration measured by the sensor should be gravity, which is known to be vertical. This information can thus be exploited to define the inclination of the sensor. The measured magnetic field can also be used to express the orientation of the sensor relative to the Earth’s magnetic field. However, in practice such ideal conditions are never obtained. Since the MIMUs are used for the purpose of characterizing human movements, accelerometers measure external acceleration induced by body segment movements in addition to the acceleration induced by gravity. In addition, magnetometers are sensitive to the ferromagnetic components located near the MIMU, which is a recurrent issue.

The classic approach to processing all of these noisy and perturbed measurements to estimate MIMU orientation is to fuse them inside a Kalman filter. In fact, Kalman filtering is largely adopted to perform data fusion from noisy measurements [8,9]. Complementary filters have been more recently proposed as an alternative to Kalman filtering [10,11]. Some studies have compared the performance of such filters with that of Kalman filters during human movement [12,13]. Since the performances were relatively similar, further studies must be performed to assess the advantages and disadvantages of these two types of filtering, by measuring various human movements in terms of duration and movement acceleration. The principal concept of Kalman filtering is to combine different sensor measurements with a dynamic model that translates the time-behavior of the state to be estimated. Despite its extensive use in MIMU processing, there is no consensus as to the structure of the Kalman filter and its settings [8]. In particular, a critical point when implementing a Kalman filter is the definition initialization of the covariance matrices that characterize mismodelling and input error from noisy sensors [14]. These covariance matrices are crucial since their poor definition can lead to inaccurate estimation as well as divergence and stability problems [15]. When applied to the orientation estimation by MIMU data, covariance matrices represent measurement noise from gyroscopes, accelerometers, and magnetometers. However, they also represent the external perturbations of acceleration and the magnetic field since, as previously mentioned, both the external acceleration experienced by the sensor, and magnetic disturbances, tend to cause errors in the estimated orientation. The critical issues of the initialization of covariance matrices are little documented [16,17] and, surprisingly, are never considered in human motion captured by MIMUs.

The aim of the present study is to propose a methodology which will identify the values of these covariance matrices that optimize orientation estimation in the context of human motion analysis.

## 2. Materials and Methods

To identify the optimal values of the covariance matrices, we proposed to explore the space of sets of values for each parameter of the covariance matrices, and to compare Kalman filter results with a reference measurement. For these purposes, we compared the orientation of a rigid object measured simultaneously by an optoelectronic system and by an MIMU. The optoelectronic system was considered in this case to be the most suitable system, due to its faculty to measure a wide range of movements in terms of acceleration and angular velocity level.

### 2.1. Experimental Procedure

**Figure 1:**
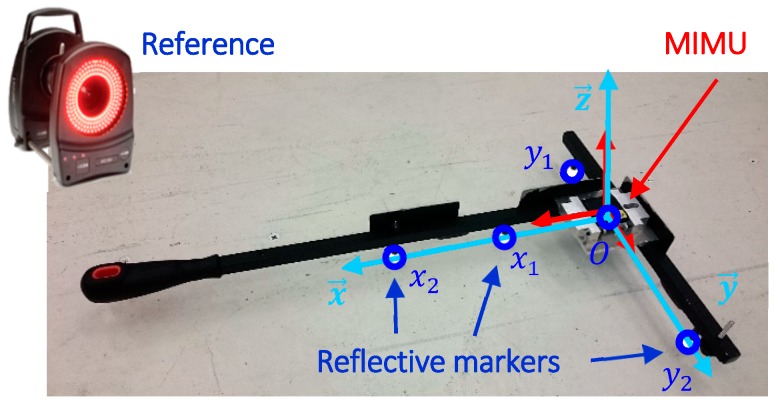
Set square equipped with both an MIMU and five reflective markers. The reflective markers can be used to define local coordinate systems associated with the set square.

The optoelectronic measurement was provided by a system of 20 cameras (Vicon Motion Systems Ltd., Oxford, UK) with a sampling frequency of 250 Hz. The object used was a set square, seen in Figure 1, the usual function of which is to calibrate the optoelectronic system. This object was particularly suitable for this study because it is pre-equipped with five reflective markers that can form a coordinate system based on the geometry of the set square. Additionally, the handle makes it easy to apply hand movements, thus limiting marker occultation.

The MIMU (Opal sensors, APDM, Portland, OR, USA) was aligned with the set square by means of a custom adapter. Sensor data (triaxial accelerometers, gyroscopes, and magnetometers) were collected at 128 Hz.

Time synchronization between both systems was ensured by different procedures. During the experiment, an external trigger executed by the launch of the MIMU system measure triggered the optoelectronic system measure. However, after resampling the measurement from the optoelectronic system by cubic interpolation at 128 Hz to be consistent with the inertial measurement, post-processing showed a time-shift between the data. Therefore, another procedure, depicted in Annex A, was applied to time-synchronize the data.

The set square was moved manually in the calibrated volume of the optoelectronic system. In order to analyze the effect of external accelerations experienced by the MIMU on covariance matrices, three different intensity levels of movement were performed on the set square. To define these three intensity levels, the set square was moved as rapidly as possible and the corresponding values were recorded. The three intensity levels were then defined by creating three equal intervals between zero and the maximal recorded values. These levels were designated by the adjectives slow, intermediate, and fast. Table 1 lists the main kinematic characteristics corresponding to each movement intensity level. Given the likely presence of static or quasi-static periods during human motion, we subsequently added a static acquisition to our protocol to analyze this particular situation.

Each movement was executed for a duration of 10 min so that the possible drift caused by the gyroscopes had time to intervene [18]. The movements were also initiated by a short static phase to estimate the initial bias of the gyroscopes, as well as the initial orientation of the set square. The nature of the imposed movements was, to the extent possible, “random” and of “globally constant” intensity. The global acceleration and angular velocity were controlled retrospectively. Five recordings for which the kinematics did not follow the same movement intensity level over the 10 min period were performed again. The movements were not collected during typical human movement, such as gait, because this could have resulted in reduced combinations of acceleration, angular velocity, and magnetic field values.

To verify the repeatability of the parameters of the covariance matrices identified, the three movements were repeated three times (on three separate days) for each of the three Opal APDM MIMUs tested. Therefore, a total of 27 motion capture sessions were made for these three sensors.

### 2.2. Gold Standard Measure

Orientation of the set square was estimated using the QUEST algorithm [19], which gives an optimal solution in a least-squares sense, to the Wahba problem [20]. The Wahba problem seeks to find a unique quaternion $\bar{q}$ that transforms all of the vectors that define the local coordinate system, based on the reflective markers seen in Figure 1, into the global coordinate system of the optoelectronic system. This algorithm makes it possible to consider all five markers for orientation, which tends to reduce measurement errors made on each of the markers. According to the QUEST algorithm, the Wahba’s problem can be formulated as:

$$\;g\left(\bar{q}\right)=\;{\bar{q}}^{T}.K.\bar{q}\;$$

where *K* is the matrix defined by:

$$\;K=\;\left[\begin{array}{cc} S-\sigma I & Z\\ Z^{T} & \sigma \end{array}\right]\;$$

with

$$\;S\;=B+B^{T}\;$$

$$\;Z=\left[\begin{array}{ccc} B_{23}-B_{32} & B_{31}-B_{13} & B_{12}-B_{21} \end{array}\right]\;$$

σ=tr[B] 

and

$$\;B\;=\underset{k}{\sum }w_{k}v_{k}^{T}\;$$

$w_{k}$ and $v_{k}$ represent the vectors defined in the local and global coordinate systems respectively. Since three markers were placed on each part of the set square, three possibilities exist to define the *x* and the *y* axis of the local coordinate system (see Figure 1 for the representation of the *x* and *y* axis). Therefore, 2 × 9 = 18 local coordinate systems can be obtained, since it is possible to define the local coordinate system by keeping the *x* axis (such that $\vec{y}\;=\;\vec{z}\;\times \;\vec{x}$) or the *y* axis (such that $\vec{x}\;=\;\vec{y}\;\times \;\vec{z}$). The same importance (and therefore the same weight in the QUEST algorithm) was given to each defined local coordinate system. The optimal quaternion corresponds to the associated normalized eigenvector, which pertains to the largest positive eigenvalues of *K* [19].

The orientation error resulting from the measured position of the reflective markers (mainly white noise) was then evaluated. First, the Allan Variance method [21] was applied to the five reflective markers during a static acquisition. For the five reflective markers, the white noise characteristic was of 5.7 μm in mean. The impact of this noise over the orientation was estimated by entering the identified white noise into simulated data that were used as input of the QUEST algorithm. This simulation enabled us to evaluate this gold-standard measurement error to 3 × 10^−30^ on the set square orientation estimate.

### 2.3. MIMU Data Processing

**Figure 2:**
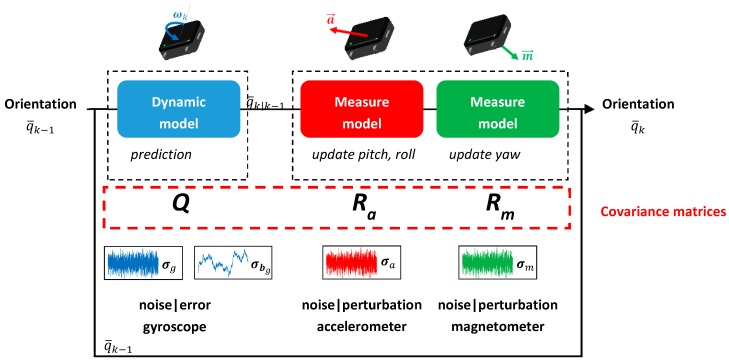
Schematic diagram of the custom Kalman filter.

The procedure was applied to three MIMUs (Opal sensors, APDM, Portland, OR, USA). Sensor data (triaxial accelerometers, gyroscopes, and magnetometers) were collected at 128 Hz and fused in a custom Kalman filter, as seen in Figure 2. According to the manufacturer, the noise density was 1.3 mm/s^2^/$\sqrt{\mathrm{Hz}}$ for the accelerometer, 2.2 mrad/s/$\sqrt{\mathrm{Hz}}\;\mathrm{for}\;\mathrm{the}\;\mathrm{gyroscope},$ and 160 nT/$\sqrt{\mathrm{Hz}}$ for the magnetometer.

As the measurement model that relates sensor data to the orientation (expressed as a quaternion) is non-linear, we built an extended Kalman filter (EKF). To facilitate quaternion computation and to lighten the state vector, we used a multiplicative indirect Kalman structure [22]. The state vector was augmented with gyroscope bias, the behavior of which was modeled as a random walk. Angular velocity was thus corrected before the numerical integration. More details about this Kalman filter can be found in Annex B.

The reference orientation of the set square obtained by the optoelectronic system was expressed in the global coordinate system of the optoelectronic system, whereas the orientation resulting from the MIMU measurement was expressed in the north-east-down (NED) geographical coordinate system (the first axis defines the northern axis, the second the eastern axis, and the last, the descending vertical). For the purpose of comparison, the orientations measured by the two systems must therefore be expressed in the same coordinate system. To this end, we identified the quaternion that represents the transformation of the optoelectronic system coordinate system into the NED coordinate system.

To identify this transformation quaternion, we exploited the measurements obtained during the experimental procedure. The identification of this rotation was carried out in two steps. Firstly, the roll-pitch components of the transformation were identified at the beginning of the acquisition during a static phase, by taking the average quaternion [23] defined as follows:

$$\;{\bar{q}}_{opto\to ned}^{roll-pitch}=\;{\bar{q}}_{opto}^{*}\;⊗\;{\bar{q}}_{MIMU}$$

Secondly, the yaw component of the transformation was identified, re-using the QUEST algorithm throughout the whole movement. This time, the optimal rotation being assessed corresponded to the transformation between the transformation ${\bar{q}}_{opto\to ned}^{roll-pitch}$ and the NED coordinate system. More details about the procedure is provided in Annex A.

### 2.4. Identification of the Kalman Parameters

As part of a multiplicative structure of the Kalman filter, the covariance matrix of the process noise was directly defined from the standard deviations characteristic of the noises of measurement [7]. The process Q and measurement $R_{a}$ and $R_{m}$ covariance matrices were then defined as follows:

$$Q=\left[\begin{array}{cc} \sigma_{g}^{2}.I_{3} & O_{3}\\ O_{3} & \sigma_{bg}^{2}.I_{3} \end{array}\right],\;R_{a}=\sigma_{a}^{2}.I_{3},\;\mathrm{and}\;R_{m}=\sigma_{m}^{2}.I_{3}$$

From this assumption, $\sigma_{g}$, $\sigma_{a},$ and $\sigma_{m}$, denote respectively the standard deviation of the gyroscope, accelerometer, and magnetometer global errors (noises, external perturbations, calibration defects etc.), while $\sigma_{bg}$ represents the standard deviation of the gyroscope bias. The four parameters that were identified will be henceforth referred to as the Kalman parameters.

**Figure 3:**
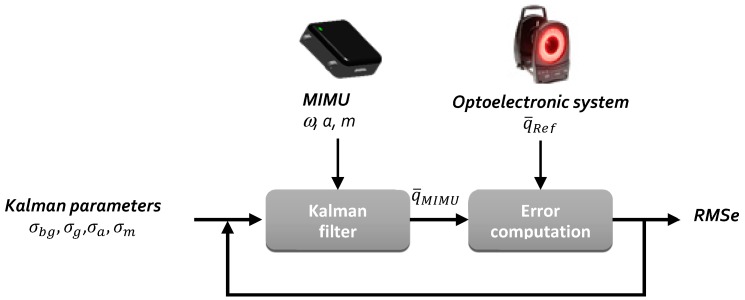
Identification process. MIMU: magneto-inertial measurement units; *RMSe*: root mean squared error.

The process used to identify the four parameters is presented in Figure 3. For each set of Kalman parameters, the orientation was computed by means of the Kalman filter. On each occasion, this orientation was compared to the orientation resulting from the optoelectronic measurement.

This comparison enabled us to calculate the quaternion orientation error as follows:

$$\;d\bar{q}={\bar{q}}_{MIMU}⊗{\bar{q}}_{ref}^{*}\;$$

This quaternion thus defined the rotation transformation from the orientation obtained with the optoelectronic system into the orientation obtained with the Kalman filter. Returning to the definition of the quaternion, we could then calculate the absolute angle corresponding to this rotation:

$$\;\theta =2.acos\left(d{\bar{q}}_{scal}\right)\;$$

Finally, the quality of orientation derived from the Kalman filter was characterized by the root mean squared error of this error angle over the 10 min of acquisition. This value, named *RMSe*, was obtained by the formula:

$$\;RMS_{e}=\sqrt{\frac{1}{N}\overset{N}{\underset{k=1}{\sum }}\theta^{2}}\;$$

where *N* represents the number of measures over the 10 min.

To reduce the possible combinations of Kalman parameters, a set of ten values per parameter was chosen. This set of parameters was initially based on the characteristic values of measurement noises identified by the Allan’s Variance method as seen in Table 2. These values were considered to be the minimal value for each Kalman parameter, because they should represent the minimum measurement errors. In fact, by definition they address only two stochastic disturbances (white noise and bias instability). So, to integrate the additional errors (other stochastic perturbations, calibration and model errors, and external perturbation), the nine other values of the set consisted of nine higher values. To explore a wide range of values, the ten values were regularly spaced on a logarithmic scale. This initial set of parameters was then refined to target the optimal parameters that were originally identified.

We proposed to identify the parameters in two steps. First, the error was evaluated only in terms of roll-pitch, the effect of the magnetometers being therefore discarded. This approach made it possible to consider only three parameters initially: *σ_g_*, *σ_bg_*, and *σ_a_*. At the end of this first step an “ideal” value of *σ_bg_* was obtained. In the second step, the complete error was analyzed by integrating the effect of the magnetometers. We then considered the three parameters *σ_g_*, *σ_a_*, and *σ_m_*. Indeed, these three parameters are very closely linked and must be computed together.

This approach was adopted to provide a reasonable duration for the identification process. For example, given that the execution of the Kalman filter on a 10 min acquisition took about 30 s, the first identification step of three *σ_g_*, *σ_bg_*, and *σ_a_* lasted around 27 × 103 × 30 = 810,000 s, which already corresponded to nine days. 

### 2.5. Optimality Criterion

For each movement, the average orientation errors on the three MIMUs tested, and on the three repetitions, were exploited. From the nine acquisitions at each intensity of movement, average and standard deviation of the *RMSe* were computed for each combination of Kalman parameters. To define the optimal values, it was proposed to consider a parameter defined by the sum of the *RMSe* and its associated standard deviation. Our optimality criterion was then to minimize this parameter. As such, this parameter takes into account not only the orientation error but also its variability during the identification process, which is more suitable for generalizing these results across more than three sensors.

### 2.6. Validation Procedure

To test the efficiency of the Kalman filter based on the optimal parameters identified, we performed a new experiment. First, we imposed on an MIMU from the same manufacturer (Opal sensors, APDM, Portland, OR, USA), that was not used during the identification procedure, a succession of movements, varying in intensity for a duration of 10 min. Then, we obtained orientations by setting the Kalman filter in five different ways. We first set the Kalman filter with the values of measurement noise identified by the Allan variance method, as seen in Table 2, then with the optimal Kalman parameters obtained for slow, intermediate, and fast movements. In addition, we implemented an adaptive Kalman filter as proposed by several authors [24,25]. In this study, we set the Kalman filter so that it selected automatically the set of parameters corresponding to the movement intensity, depending on the instantaneous acceleration and rotational speed. To prevent the Kalman filter from “oscillating” between two intensity modes, we defined a minimum duration of 10 s between each mode change. We will henceforth refer to this setting as “the adapted Kalman filter”.

The error angle between the orientations obtained with each of the five different settings of the Kalman filter and the orientation obtained with the optoelectronic system was computed as well as the *RMSe* as previously depicted over the 10 min of acquisition.

The orientation error for the orientation obtained from the manufacturer algorithm was also computed. According to the manufacturer, their algorithm uses a state space model with a causal Kalman Filter. Different settings are possible. We chose the “Variable Weight Magnetometer” setting since according to the information provided by the manufacturer “it is the best choice for an environment similar to where the field calibration was last performed, and where having an absolute heading reference is desired”, which was the case here.

## 3. Results

### 3.1. Identification of the Kalman Parameters

**Figure 4:**
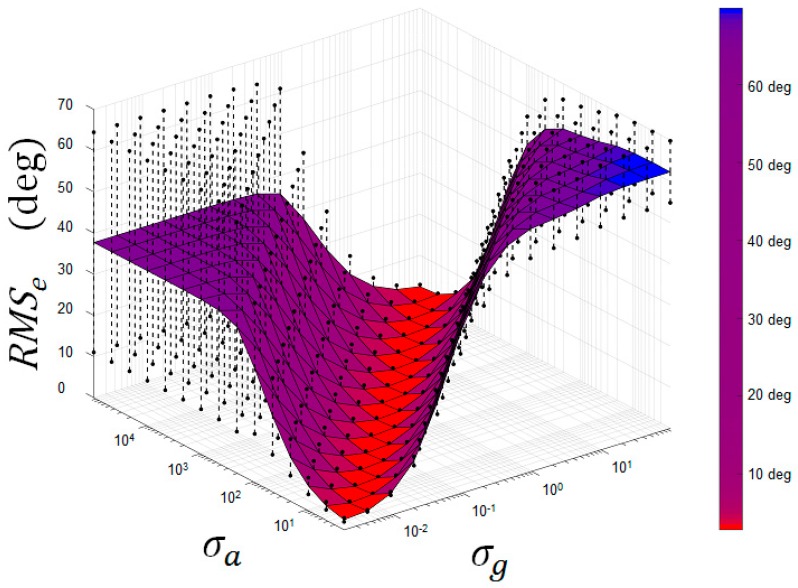
*RMSe* and standard error (black dots) of the *RMSe* between the orientation estimated with the optoelectronic system and the Kalman filter, depending on the values of Kalman parameters *σ_g_* and *σ_a_* for the intermediate intensity movements.

Beyond identifying optimal values to assign to Kalman parameters *σ_g_*, *σ_bg_*, *σ_a_*, and *σ_m_*, the analysis of the results revealed interesting information concerning the behavior of the Kalman filter. For instance, Figure 4 represents, for the intermediate intensity movements, the *RMSe* translating orientation error between the orientation estimated with the optoelectronic system and that obtained with the Kalman filter, depending on the values of the Kalman parameters *σ_g_* and *σ_a_*. Most importantly, Figure 4 shows that a bad parameter choice can induce a huge error (up to 65° for this movement), which confirms that appropriate selection of the Kalman parameters is essential.

The area on the extreme right of the figure corresponds to high values assigned to *σ_g_* and low values assigned to *σ_a_*. In other words, these values force the Kalman filter to give maximum importance to the measurement resulting from the accelerometers, and to neglect the measurement from the gyroscopes. Conversely, the area on the far left corresponds to low values assigned to *σ_g_* and high values assigned to *σ_a_*. In other words, these values force the Kalman filter to give maximum importance to measurement from gyroscopes, and to neglect the measurement resulting from the accelerometers. These two areas usually lead to significant errors. Indeed, the error observed in the right-hand area translates into an error in orientation estimation due to external accelerations experienced by the MIMUs, while the error observed in the left-hand area corresponds to the drift phenomenon resulting from the numerical integration of noisy angular velocity. The zone containing the optimal values is therefore located between these two particular areas, since it reflects a compromise between the gyroscope measurement and the measurement resulting from the accelerometers.

Table 3 provides the optimal values for each Kalman parameter and the *RMSe* obtained for these values. These results confirm that the Kalman parameters are related to movement intensity. It is especially noted that *σ_a_* is proportional to movement intensity (directly related with the external acceleration). These results also highlight the particular behavior of static condition because of which the Kalman filter must grant much less importance to gyroscopes (a hundred times less) in order to counteract bias instability.

It can also be seen that the *RMSe* could not be reduced to under 13.7° in fast movements.

### 3.2. Results from the Validation Procedure

**Figure 5:**
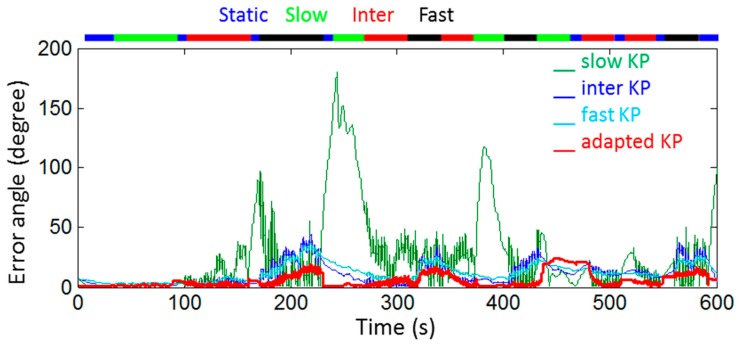
Orientation errors computed during a 10 min movement for orientations obtained with four different settings of the Kalman filter, namely with adapted Kalman parameters and with Kalman parameters identified respectively on slow, intermediate, and fast movements. The orientation obtained with the optoelectronic system served as reference. The color bar at the top of the figure represents the intensity level recorded during the movement.

Figure 5 represents, over the whole movement, the orientation error computed between the orientation obtained with the optoelectronic system and those obtained with the four different settings of the Kalman filter based on the identified Kalman parameters. Because the orientation obtained with the Kalman filter based on the noise identified by the Allan’s Variance method was particularly bad, for greater clarity we chose not to represent it in Figure 5.

**Figure 6:**
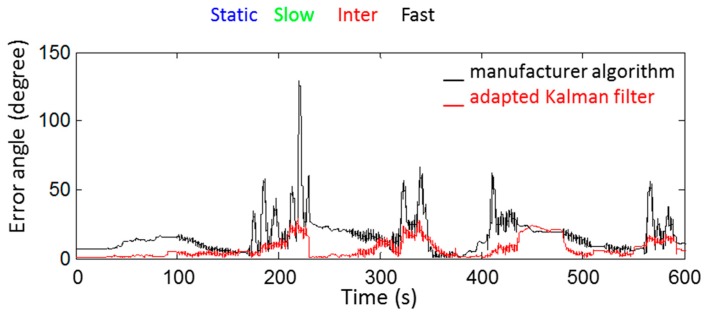
Orientation errors computed during the same 10 min movement for orientations obtained with adapted Kalman parameters and with the manufacturer algorithm. The orientation obtained with the optoelectronic system served as reference. The color bar at the top of the figure represents the intensity level recorded during the movement.

The *RMSe* computed during the whole movement was 10° for the orientation obtained with the adapted Kalman parameters, and 46°, 16°, and 15° for the orientation obtained with the Kalman parameters identified respectively on slow, intermediate, and fast movements. The *RMSe* was 95° for that obtained with the Kalman parameters based on the noise identified by the Allan’s Variance method. With the manufacturer algorithm, the *RMSe* computed during the whole movement was 20° for the orientation obtained, as seen in Figure 6. 

## 4. Discussion

In this study, a method for initializing covariance matrices has been proposed by comparing the inertial measurement with an optoelectronic measurement used as reference in the context of human motion analysis.

Results highlight the need to select the Kalman parameters required to define these covariance matrices rigorously, otherwise the performance of the Kalman filter can be seriously altered. This identification is even more important in that the optimal values obtained are relatively different from the quantities estimated by the Allan Variance method. Moreover, with the Kalman filter set to the quantities estimated by the Allan Variance method, considerable orientation error was found. The main advantage of the proposed approach is that it is global. Therefore, errors taken into account gather stochastic errors as well as behaviors not taken into account by the calibration models, or by the modeling carried out within the Kalman filter.

Our results clearly show that the optimal Kalman parameter values depend on movement intensity, which is of great importance for applications requiring human movement characterization. We proposed in the present paper to build a Kalman filter such that it automatically selected the set of Kalman parameters corresponding to the movement intensity, depending on the instantaneous acceleration and rotational speed. With such an adaptation, during a 10 min movement, orientation *RMSe* was reduced to 10° compared to the 15° obtained with the Kalman filter set to the optimal Kalman parameters obtained for fast movements. The remaining 10° of orientation *RMSe* can probably be mainly imputed to persistent problems that affect orientation estimation by MIMU for fast motion. Indeed, during the identification procedure, the difference between orientations obtained with the optoelectronic system and with our Kalman filter—even optimized—remained above 10° for fast movements. In that case, the optimal behavior of the Kalman filter leads us to place much more importance on gyroscopes than on accelerometers. Therefore, the drift phenomenon resulting from the numerical integration of noisy angular velocity cannot be avoided. This underlines the need for less noisy gyroscopes [26]. We have to admit that MIMUs nowadays include gyroscopes of higher quality than those present in the MIMUs used in this study. Noise density and bias stability of the gyroscopes are in fact greatly reduced in the new generation of MIMUs. For instance, the gyroscope noise density announced by the manufacturer was 0.05°/s/sqrt(Hz) for the version of the sensors used in the present study, whereas it is of 0.025°/s/√Hz for the latest version of these sensors [27]. Therefore, better results can be expected in the future. Moreover, our protocol designed a fast motion with 4 g acceleration and 700°/s angular velocity during 10 min. Such intensity is hardly to be expected during 10 min of human movement.

To test the benefits of the proposed method on MIMUs of different quality, and from a different manufacturer, we applied this methodology to another MIMU (MicroStrain 3DM-GX4-25). The corresponding results are presented in Appendix A. For this tactical-grade MIMU, the orientation error was also notably reduced (from 0.8 to 0.4°) despite the already small orientation error obtained with the manufacturer algorithm also based on Kalman filter, as seen in Table A1. Therefore, we are confident that the present methodology can be beneficial to all sorts of MIMUs in the context of human movement analysis.

Furthermore, let us notice that it is not straightforward to compare orientation errors obtained in the present study with orientation errors mentioned in previous studies. Not only were MIMUs used in the studies from different manufacturers and, as such, have not identical technical features, but also movement characteristics used to validate the methodology might have been different in terms of duration, linear acceleration, and angular velocity. Unfortunately, in previous studies movement characteristics are seldom fully documented. In the present paper, we obtain an orientation error of 1.5° and 2.9° for movements performed at respectively slow and intermediate intensity movement. Our results are close to Sabatini’s results who presented an orientation error of 1.63° with a new extended Kalman Filter [8]. However, as this previous study did not provide information regarding the movement intensity level, additional interpretation of the differences between the results from Sabatini’s study and the present one would remain speculative. 

Our present study focused on Kalman filter. Recent publications showed complementary filters as a promising alternative for fusing inertial data to obtain orientation [10,11]. For instance, a recent paper has tested an improved explicit complementary filter that uses only acceleration and gyroscope data as inputs [28]. The orientation error for the yaw angle remains close to 1° but the nature of the movement tested is here again not known. Further investigations should then be conducted in order to assess thoroughly the advantages and disadvantages of complementary filters comparatively to Kalman filters.

Our proposed method has the disadvantage of being painstaking to achieve, not only in terms of experimentation but also in terms of computation, since many combinations of Kalman parameters have to be tested. To speed up the process, an optimization algorithm could be implemented to run the Kalman filter a small number of times. Other studies of minimal movement duration should be also performed to reduce the experimentation time. The proposed methodology required an optoelectronic system to provide the reference orientation. Robotic devices can probably be used to substitute this optoelectronic system and also the tester who is required to move the set square.

In the present paper, the identification procedure has been proposed from the study of three different movement intensities. The behavior of the Kalman filter in these situations should be considered in any study of human movement that is likely to lead to intermediate or mixed intensity movements. It is possible at first to simply exploit the Kalman parameters corresponding to the closest intensity tested, as proposed in the validation section of the present study. However, to go further, a continuous approach to Kalman parameter management could be considered. Indeed, the analysis of the roll-pitch component of the error showed that the optimal behavior of the Kalman filter was obtained by respecting a certain ratio between *σ_a_*/*σ_b_* function of movement intensity. A procedure that defines the evolution of this ratio according to the measured accelerations could then be proposed. We have in this study considered Kalman parameters common to all three Opal APDM MIMUs tested. However, each MIMU is slightly different, such that the assignment of specific parameters to each sensor could be advantageous. Such a choice would, however, require the reproduction of this identification process for all MIMUs.

One can also notice that the movements performed were not “natural” human movements, such as walking. Therefore, the real benefits of the presented method for human movement analysis must be further probed. 

Finally, only the influence of movement intensity on the Kalman parameters has been tested. Additional, similar studies could also be conducted to specify the influence of magnetic perturbations on the optimal Kalman parameters.

## 5. Conclusions

The method presented provides a unique solution to the problem of identifying the optimal initial covariance matrices values for Kalman filtering. With these optimized initial covariance matrices, the orientation estimation was greatly improved. However, the methodology should be improved to reduce the duration of the whole process.

## Figures and Tables

**Table 1:** Main kinematic characteristics of the imposed movements.

Intensity	Slow	Intermediate	Fast
Acceleration (g)	0.03 ± 0.02	0.7 ± 0.5	4 ± 2
Angular velocity (°/s)	40 ± 20	300 ± 150	700 ± 400

**Table 2:** Values of measurement noise identified by the Allan’s Variance method.

*σ_bg_*	*σ_g_*	*σ_a_*	*σ_m_*
3 × 10^−3^ rad/s	1 × 10^−3^ rad/s	4 × 10^−3^ m/s^2^	0.2 µT

**Table 3:** Identified Kalman parameters and corresponding *RMSe*.

	Static	Slow	Intermediate	Fast
*σ_bg_* (rad/s)	10^−4^	10^−4^	10^−3^	3 × 10^−5^
*σ_g_* (rad/s)	0.1	10^−3^	10^−2^	6 × 10^−3^
*σ_a_* (m/s^2^)	0.2	0.2	8	10
*σ_m_* (µT)	10	1.5	4	45
*RMSe* (degree)	0.08 ± 0.01	1.5 ± 0.2	2.9 ± 0.3	13.7 ± 5.3

**Figure A1:**
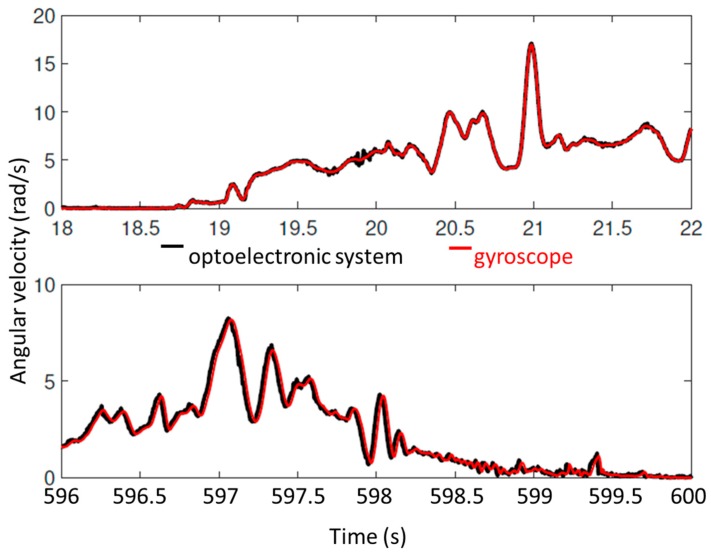
Comparison of the angular velocity obtained from the MIMU (in red) with that obtained from the optoelectronic system (in black) at the beginning of the 10 min acquisition (at the top) and at end (at the bottom).

**Figure A2:**
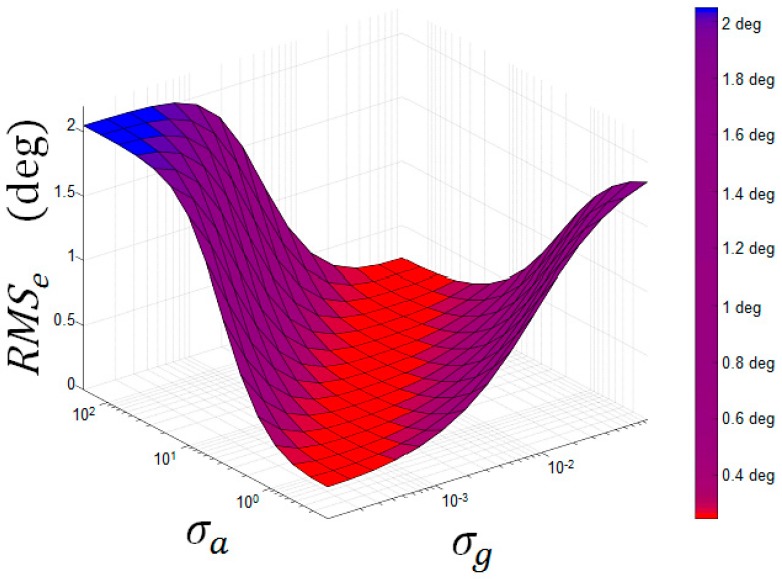
*RMSe* between the orientation estimated with the optoelectronic system and the Kalman filter depending on the values of Kalman parameters *σ_g_* and *σ_a_* for the slow intensity movement.

## Appendix A. Synchronization of the MIMUs and Optoelectronic Systems

### Appendix A.1. Temporal Synchronization

A process of synchronization of the two systems was initially planned by managing the triggering of the optoelectronic system from a rising edge executed by the MIMU measurement system. However, the effectiveness of this method was not satisfactory, because a significant time shift remained between the two measures.

We then implemented an optimization process to minimize the gap between the angular velocity measured by the two systems with a least squares approach. For the optoelectronic system, the angular velocity expressed in the local coordinate of the set square was obtained by discrete time-derivation of the quaternion angle as follows:

$$\;\theta =2\times acos\left(q_{scal}\right)\;$$

and then

$$\;\omega =\;\frac{\Delta \theta }{\Delta t}\;$$

Nevertheless, even after making this correction, a time lag appeared at the end of the acquisition. As illustrated by Figure A1, the two curves were well synchronized at the beginning of the acquisition but were no longer so at the end of the acquisition (i.e., after 10 min of measurement). This systematic observation revealed a problem of temporal proportionality between the two measurement systems.

A new optimization process was then implemented to identify the temporal homothety to apply to the optoelectronic data. Once applied, the synchronization of data throughout the acquisition was obtained. According to this optimization process, the temporal proportionality coefficient to be applied was systematically 0.003% or 30 ppm. This value shows that the progressive desynchronization observed was very weak. Indeed, this corresponds to an offset of 18 ms after an acquisition of 10 min, which is then equivalent to just over two points on a signal sampled at 128 Hz.

We attributed a higher confidence to the MIMU sensors, because their data are time-stamped, which is not the case for the data from the optoelectronic acquisition. Indeed, for the optoelectronic system, the time stamps are just deduced from the sampling frequency, without any control of the actual measurement times. This hypothesis was confirmed during the equivalent analysis conducted with another MIMU, the MicroStrain 3DMGX4-25, which had the same result. This result implies that characterization of the time of the optoelectronic measurement does not seem to be without fault.

### Appendix A.2. Identification of the Quaternion Representing the Transformation of the Optoelectronic System Coordinate System into the MIMU Coordinate System

This procedure was carried out in two steps. Firstly, the pitch and roll components were identified and then the yaw component of the transformation between the optoelectronic coordinate system and the MIMU coordinate system.

For that purpose, during a static phase during which the set square was lying on the floor, at each time-step, the transformation quaternion between the two coordinate systems was identified according to the formula:

$$\;{\bar{q}}_{opto\to ned}^{roll-pitch}=\;{\bar{q}}_{opto}^{*}\;⊗\;{\bar{q}}_{MIMU}\;$$

Then, the average quaternion was computed according to the Markley et al. method [23]. One could notice that this transformation quaternion had mainly a roll component of 180°. In fact, the MIMU coordinate system is the north-east-down (NED) coordinate system. Therefore, its *z* axis is vertical, pointing downward, whereas the *z* axis of the optoelectronic coordinate system, which is perpendicular to the calibration wand placed on the floor during the calibration procedure, is almost vertical, pointing upward.

An intermediate coordinate system equivalent to the NED coordinate system in terms of pitch and roll, but different in terms of yaw, was then obtained.

During the movement, the quaternions obtained with the optoelectronic system were then expressed in this temporary coordinate system as follows:

$${\bar{q}}_{opto}^{tmp}=\;{\bar{q}}_{opto}⊗\;{\bar{q}}_{opto\to ned}^{roll-pitch}\;$$

The same procedure was not used during the second step. Indeed, the yaw component obtained from inertial measure is the component that suffers the most from inaccuracy. Moreover, the static phase being short, we preferred to estimate the yaw component during the whole movement. For these different reasons, we used a QUEST algorithm and only the magnetic field measure.

The QUEST algorithm seeks the optimal rotation in the least squares sense [29]. Here, we expressed two vectors in the NED coordinate systems:

$$r_{1}=h\;\mathrm{and}\;r_{2}=\left[\begin{array}{c} 0\\ 0\\ 1 \end{array}\right]\;$$

*h* being the magnetic field.

Two other vectors were expressed in the intermediate coordinate system:

$$s_{1}=\tilde{m}\;\mathrm{and}\;s_{2}=\left[\begin{array}{c} 0\\ 0\\ 1 \end{array}\right]\;$$

$\tilde{m}$ being the magnetic field measured by the inertial sensor and expressed in the intermediate coordinate system. According to the formula:

$$\;\tilde{m}={\bar{(q}}_{opto}⊗{\bar{q}}_{opto\to ned}^{roll-pitch}{)}^{*}⊗\;m\;⊗\;{\bar{q}}_{opto}⊗\;{\bar{q}}_{opto\to ned}^{roll-pitch}\;$$

The QUEST method is then used to find the attitude matrix A, such that:

$$Ar_{1}=\;s_{1}\;\mathrm{and}\;Ar_{2}=\;s_{2}\;$$

The quaternions obtained through the QUEST method are then averaged to obtain ${\bar{q}}_{opto\to ned}^{y}$.

Quaternions measured from the optoelectronic system and expressed in the NED coordinate system can then be obtained as follows:

$$\;{\bar{q}}_{opto}^{Rned}=\;{\bar{q}}_{opto}^{Ropto}⊗\;{\bar{q}}_{opto\to ned}^{roll-pitch}⊗\;{\bar{q}}_{opto\to ned}^{y}\;$$

## Appendix B. Indirect Kalman Filter Used in the Study

The Kalman filter proposed in this study is partially based on a filter described by Trawny and Roumeliotis [22]. The update is based on the propositions made by Suh [25].

### Appendix B.1. Attitude Prediction

In this model, the state x is given by the quaternion and the gyroscope bias:

$$\;x=\;\left[\begin{array}{c} \bar{q}\\ b \end{array}\right]\;$$

The state estimate is predicted at time-step *k* by:

$$\;x_{k}=\;F_{k}.x_{k-1}+v_{k-1}\;$$

$F_{k}$ being the state transition model at time-step *k* and $v_{k-1}$ the process noise at time-step (*k* − 1)

The gyroscope bias is represented as a random walk process; we can predict the current gyroscope bias such as:

$$\;{\hat{b}}_{k|k-1}={\hat{b}}_{k-1}\;$$

A first estimation of the angular velocity is then provided by:

$${\hat{\omega }}_{k|k-1}={\hat{\omega }}_{k}^{m}-{\hat{b}}_{k|k-1}\;$$

${\hat{\omega }}_{k}^{m}$ being the angular velocity measured by the gyroscope at time-step *k*.

The actual quaternion can be predicted based on this angular velocity and the quaternion at the previous time-step according to a first-order quaternion integrator:

$$\;{\hat{\bar{q}}}_{k|k-1}=\;\left[\exp\left(\frac{1}{2}.\Omega (\bar{\omega }).T\right)+\frac{1}{48}\left(\Omega \left({\hat{\omega }}_{k|k-1}\right).\Omega ({\hat{\omega }}_{k-1})-\Omega ({\hat{\omega }}_{k-1}).\Omega ({\hat{\omega }}_{k|k-1})\right)T^{2}\right].{\bar{\hat{q}}}_{k-1}\;$$

with $\bar{\omega }$ the mean angular velocity defined by:

$$\;\bar{\omega }=\frac{{\hat{\omega }}_{k|k-1-{\hat{\omega }}_{k-1}}}{2}\;$$

We consider an indirect approach; the prediction is then performed with the error state equation defined by the orientation error δθ and the bias error Δb as:

$$\;\tilde{x}=\;\left[\begin{array}{c} \delta \theta \\ \Delta b \end{array}\right]\;$$

The dynamic model is here given by:

$$\;\dot{\tilde{x}}=F_{c}.\tilde{x}+G_{c}.v\;$$

with $F_{c}$ and $G_{c}$, *v* the process noise formulated as:

$$F_{c}=\left[\begin{array}{cc} -\left[\hat{\omega }\times \right] & -I_{3}\\ 0_{3} & 0_{3} \end{array}\right]\;G_{c}=\;\left[\begin{array}{cc} -I_{3} & 0_{3}\\ 0_{3} & I_{3} \end{array}\right]\;v=\left[\begin{array}{c} v_{g}\\ v_{b_{g}} \end{array}\right]\;$$

$\left[\hat{u}\times \right]$ is the preproduct matricial product of a vector *u.*

The corresponding discrete state transition matrix *Φ* is given by:

$$\Phi \;=\left[\begin{array}{cc} \Theta & \Psi \\ 0_{3} & I_{3} \end{array}\right]\;$$

with

$$\Theta \;=\cos\left(\left|\hat{\omega }\right|T\right).\;I_{3}-\sin\left(\left|\hat{\omega }T\right|\right).\left[\frac{\hat{\omega }}{\left|\hat{\omega }\right|}\times \right]+\left(1-cos\left(\left|\hat{\omega }\right|T\right)\right).\left[\frac{\hat{\omega }}{\left|\hat{\omega }\right|}\frac{{\hat{\omega }}^{T}}{\left|\hat{\omega }\right|}\right]\;$$

and

$$\Psi \;=-\;I_{3}.T+\frac{1}{{\left|\hat{\omega }\right|}^{2}}\left(1-\cos\left(\left|\hat{\omega }\right|T\right)\right).\left[\hat{\omega }\times \right]-\;\frac{1}{{\left|\hat{\omega }\right|}^{3}}\left(\left|\hat{\omega }\right|T-\sin\left(\left|\hat{\omega }\right|T\right)\right).{\left[\hat{\omega }\times \right]}^{2}\;$$

The noise covariance matrix is defined by:

$$\;Q_{c}=\;\left[\begin{array}{cc} \sigma_{g}^{2}.I_{3} & 0_{3}\\ 0_{3} & \sigma_{b_{g}}^{2}.I_{3} \end{array}\right]\;$$

which becomes in a discrete case:

$$\;Q_{d}=\;\left[\begin{array}{cc} Q_{11} & Q_{12}\\ Q_{12} & Q_{22} \end{array}\right]\;$$

with

$$\;Q_{11}=\;\sigma_{g}^{2}.T.I_{3}+\sigma_{b_{g}}^{2}.(I_{3}.\frac{T^{3}}{3}+\frac{\frac{{\left(\left|\hat{\omega }\right|T\right)}^{3}}{3}+2\sin\left(\left|\hat{\omega }\right|T\right)-2\left|\hat{\omega }\right|T}{{\left|\hat{\omega }\right|}^{5}}.\;{\left[\hat{\omega }\times \right]}^{2}\;$$

$$\;Q_{12}=\;-\sigma_{b_{g}}^{2}.(I_{3}.\frac{T^{2}}{2}+\frac{\left|\hat{\omega }\right|T-\sin\left(\left|\hat{\omega }\right|T\right)}{{\left|\hat{\omega }\right|}^{3}}.\;\left[\hat{\omega }\times \right]+\frac{\frac{{\left(\left|\hat{\omega }\right|T\right)}^{2}}{2}+\cos\left(\left|\hat{\omega }\right|T\right)-1}{{\left|\hat{\omega }\right|}^{4}}.\;{\left[\hat{\omega }\times \right]}^{2}\;$$

$$\;Q_{22}=\;\sigma_{b_{g}}^{2}.T.I_{3}\;$$

The propagation of the state covariance matrix is then:

$$\;P_{k|k-1}=\Phi_{k}.P_{k-1}.\Phi_{k}^{T}+Q_{dk-1}\;$$

### Appendix B.2. Update

The update is performed in two steps as proposed by Suh [25]. The first step modifies the pitch and roll component by the use of the acceleration [25] (*k* is omitted for a sake of clarity):

$$\;z_{a}=H_{a}.x+n_{a}\;$$

The state vector is then only defined by:

$$\;z_{a}=a\;$$

and the covariance matrix is written:

$$\;R_{a}=\;\sigma_{a}^{2}.I_{3}\;$$

By adaptation of the proposal made by [22], we obtain:

$$\;H_{a}=\left[\begin{array}{cc} \left[{\hat{z}}_{a}\times \right] & 0_{3} \end{array}\right]\;$$

where ${\hat{z}}_{a}$ is the theoretical acceleration computed from the predicted orientation:

$$\;\left[\begin{array}{c} {\hat{z}}_{a}\\ 0 \end{array}\right]={\bar{q}}_{k|k-1}⊗\left[\begin{array}{c} -g\\ 0 \end{array}\right]⊗{\bar{q}}_{k|k-1}^{*}\;$$

The residual is given by:

$$\;r_{a}=z_{a}-{\hat{z}}_{a}\;$$

and the associate covariance matrix is provided by:

$$\;S_{a}=\;H_{a}.P_{k|k-1}H_{a}^{T}+R_{a}\;$$

The Kalman gain is then computed as:

$$\;K_{a}=\;P_{k|k-1}.H_{a}^{T}.S_{a}^{-1}\;$$

The correction is then obtained by:

$$\;\Delta {\hat{x}}_{a}=\;\left[\begin{array}{c} \delta {\hat{\theta }}_{a}\\ \Delta {\hat{b}}_{a} \end{array}\right]=\;K_{a}.r_{a}\;$$

The corrective quaternion is then:

$$\;\delta {\hat{\bar{q}}}_{a}=\left[\begin{array}{c} \frac{1}{2}\delta {\hat{\theta }}_{a}\\ \sqrt{1-\left|\frac{1}{2}\delta {\hat{\theta }}_{a}\right|} \end{array}\right]\;$$

and the quaternion update:

$$\;{\hat{\bar{q}}}_{a,k}=\;\delta {\hat{\bar{q}}}_{a}⊗{\hat{\bar{q}}}_{k|k-1}\;$$

Now, the gyroscope bias is updated as following:

$$\;{\hat{b}}_{a,k}={\hat{b}}_{k|k-1}+\Delta {\hat{b}}_{a}\;$$

Then the angular velocity estimated can be improved by:

$$\;{\hat{\omega }}_{a,k}=\omega_{k}^{m}-{\hat{b}}_{a,k}\;$$

The covariance matrix of the state vector is also updated by:

$$\;P_{a,k}=P_{k|k-1}-K_{a}.H_{a}.P_{k|k-1}\;$$

The second step proposes a yaw correction based on the magnetometer measure *m*:

$$\;z_{m}=H_{m}.x+n_{m}\;$$

The state vector is only defined by:

$$\;z_{m}=m\;$$

the covariance matrix by:

$$\;R_{m}=\;\sigma_{m}^{2}.I_{3}\;$$

and the measurement matrix by:

$$\;H_{m}=\left[\begin{array}{cc} \left[{\hat{z}}_{m}\times \right] & 0_{3} \end{array}\right]\;$$

where ${\hat{z}}_{m}$ is the theoretical magnetic field computed from the orientation previously predicted:

$$\;\left[\begin{array}{c} {\hat{z}}_{m}\\ 0 \end{array}\right]={\bar{q}}_{k|k-1}⊗\left[\begin{array}{c} h\\ 0 \end{array}\right]⊗{\bar{q}}_{k|k-1}^{*}\;$$

The residual is then:

$$\;r_{m}=z_{m}-{\hat{z}}_{m}\;$$

Suh proposes to apply this update only to the quaternion and not to the gyroscope bias by simplifying the state covariance matrix:

$$\;P_{m,k}=\left[\begin{array}{cc} P_{a,k\left(1:3,1:3\right)} & 0_{3,6}\\ 0_{6,3} & 0_{6,6} \end{array}\right]\;$$

After that, the residual covariance matrix can be computed as:

$$\;S_{m}=\;H_{m}.P_{m,k}H_{m}^{T}+R_{m}\;$$

To limit the effect of the correction only to the yaw component, Suh [25] proposed to modify the computation of the gain as following:

$$\;K_{m}=\left[\begin{array}{cc} r_{3}.r_{3}^{T} & 0_{3,6}\\ 0_{6,3} & 0_{6,6} \end{array}\right]P_{m,k}.H_{m}^{T}.S_{m}^{-1}\;$$

The correction is then:

$$\;\Delta {\hat{x}}_{m}=\;\left[\begin{array}{c} \delta {\hat{\theta }}_{m}\\ 0_{3,1} \end{array}\right]=\;K_{m}.r_{m}\;$$

the new quaternion update:

$$\;{\hat{\bar{q}}}_{k}=\;\delta {\hat{\bar{q}}}_{m}⊗{\hat{\bar{q}}}_{a,k}\;$$

and the new updated covariance matrix

$$\;P_{k}=P_{a,k}-K_{m}.H_{m}.P_{a,k}\;$$

## Appendix C. Application of the Methodology to a MIMU of Tactical Grade

The same procedure was applied to another MIMU (MiscroStrain 3DM-GX4-25).

The good performance of this MIMU could be attested thanks to its bias instability assessed with the Allan variance method as seen in Table A1. The bias instability was of 10^−4^ rad/s, which is below 1.45 × 10^−4^ rad/s, the threshold under which a MIMU is characterized as being “tactical grade” [30].

The identification procedure could only be applied to slow intensity movements since the maximum angular velocity measured by this MIMU is 300°/s, which represents the mean value for the category “intermediate” intensity movements.

**Table A1:** Values of measurement noise identified by the method of the Allan variance and Kalman parameters identified for slow movements.

	*σ_bg_* (rad/s)	*σ_g_* (rad/s)	*σ_a_* (m/s^2^)	*σ_m_* (µT)
Allan’s variance	1 × 10^−4^	1 × 10^−3^	2 × 10^−2^	0.6
Identification	0	2 × 10^−4^	1	4

As for the APDM MIMUs, Figure A2 depicts that the optimal behavior of the Kalman filter was obtained by respecting a certain ratio between *σ_a_*/*σ_b_*.

The values of the Kalman parameters identified with the methodology presented in the paper are presented in Table A1. Once again, one can notice that the parameters identified are notably different from those estimated with the Allan variance method.

Table A2 presents the orientation error in terms of root mean squared error between the orientation obtained with the optoelectronic system and the orientation obtained (a) with the manufacturer algorithm (b) with our Kalman filter set with the parameters identified with the methodology described in the paper.

**Table A2:** Orientation error for slow intensity movement.

	*RMSe* (degree)
Manufacturer algorithm	0.8
Kalman filter set with identified parameters	0.4

According to this table, the orientation error resulting from the manufacturer algorithm, which was small, could nevertheless be divided by two with the Kalman filter and identification methodology proposed in the paper.
